# Clinical Characteristics and Risk Factors for Pertussis in Children

**DOI:** 10.7759/cureus.84485

**Published:** 2025-05-20

**Authors:** Amira Elhoufey

**Affiliations:** 1 Department of Community Health Nursing, Alddrab University College, Jazan University, Jazan, SAU

**Keywords:** clinical features, incomplete immunization, pediatrics, pertussis, risk factors

## Abstract

Aim: This study aimed to investigate the clinical characteristics and risk factors associated with pertussis in children.

Methods: A retrospective analysis was conducted on the clinical data, risk factors, and laboratory indices of 100 children hospitalized with pertussis (pertussis group) and 100 children hospitalized with cough (control group) from January to December 2024.

Results: The serum globulin and immunoglobulin M (IgM) levels in the pertussis group were significantly lower than those in the control group. The mean hospital stay length, C-reactive protein (CRP), and white blood cell (WBC) count were significantly higher in the pertussis group. The rates of delayed immunization and non-immunization with the diphtheria, tetanus, and pertussis (DTP) vaccine were significantly higher in the pertussis group compared to the control group.

Conclusion: Delayed immunization and non-immunization with the DPT vaccine were identified as risk factors for pertussis infection. Timely vaccination with the pertussis vaccine and isolation from individuals suspected of carrying the infection are crucial for reducing pertussis incidence in infants and young children.

## Introduction

Pertussis is an acute respiratory infectious disease caused by *Bordetella pertussis* that remains a significant public health concern globally. Despite high vaccination coverage in many countries, recent years have seen a resurgence of pertussis in both developed and developing regions, with periodic epidemic peaks every 3-5 years. The World Health Organization (WHO) estimates that there are approximately 24.1 million cases of pertussis annually, with 160,700 deaths among children under the age of five due to the disease, particularly in low- and middle-income countries where immunization rates remain suboptimal [1,2]. Factors contributing to its persistence include waning immunity from acellular vaccines, underreporting, and delayed diagnosis. Insufficient vaccine immunity, atypical clinical manifestations, limitations of pathogen detection methods, and insufficient attention from medical staff have led to the spread and prevalence of pertussis, as well as a high incidence of complications, which threaten the physical and mental health of children and even their lives [1,2]. This study aimed to highlight the clinical characteristics of pertussis and analyze its risk factors in a cohort of pediatric patients in Assiut University Hospital, Assiut, Egypt.

## Materials and methods

Ethical approval and consent statement

The study was approved by the Institutional Review Board (IRB) of Assiut University Hospitals (IRB approval number: 025-300518). Informed consent was waived due to the retrospective nature of the study and the use of anonymized data. All patient information was handled confidentially, and data were used solely for research purposes.

Study design and period

This retrospective cohort study analyzed clinical and laboratory data of pediatric patients hospitalized at Assiut University Hospitals between January and December 2024. The study period was selected based on the availability of complete medical records and diagnostic test results for *Bordetella pertussis*.

Sample size and sampling method

The study aimed to compare clinical and laboratory parameters (e.g., immunoglobulin M (IgM), C-reactive protein (CRP), and vaccination status) between groups. Based on prior research [1,3], medium to large effect sizes (Cohen's d ≥ 0.5) were anticipated for differences in immune markers and vaccination status. A sample size of 100 per group provides >80% power to detect such effects at α = 0.05 using standard tests (e.g., t-test and Chi-square test).

Participants

A total of 200 children aged one month to six years were included and divided into two groups: pertussis group (100 children confirmed positive for *Bordetella pertussis* via polymerase chain reaction (PCR) testing) and control group (100 children with cough symptoms but negative PCR results for *Bordetella pertussis*). Participants were selected through consecutive sampling of eligible cases during the study period.

Inclusion criteria

Participants must be ≤18 years. For the patient group, there must be a clinical diagnosis of pertussis based on previous criteria [2,3]. Diagnosis of pertussis was confirmed using a commercially available real-time polymerase chain reaction (PCR) assay targeting the IS481 repetitive sequence specific to *Bordetella pertussis*. Nasopharyngeal swabs were collected from all participants, and DNA extraction was performed using the QIAamp DNA Mini Kit (Qiagen, Hilden, Germany), according to the manufacturer's instructions. Real-time PCR was carried out using the Applied Biosystems 7500 Fast Real-Time PCR System (Thermo Fisher Scientific, Waltham, MA) with a detection threshold set at ≤35 cycles. Positive and negative controls were included in each run to ensure accuracy and prevent cross-contamination. For the control group, participants must have cough symptoms without evidence of *Bordetella pertussis* infection. Complete clinical and laboratory records must be available.

Exclusion criteria

Those with incomplete medical records, comorbidities (e.g., chronic respiratory diseases, asthma, and immunodeficiency), and co-infection with other pathogens (e.g., *Mycoplasma pneumoniae *and respiratory syncytial virus) were excluded.

Data collection

Clinical data were extracted from electronic medical records, including demographic information (age and sex), vaccination history (diphtheria, tetanus, and pertussis (DTP) status: fully vaccinated, delayed vaccination, or unvaccinated), duration of hospitalization, and laboratory parameters (C-reactive protein (CRP), white blood cell (WBC) count, immunoglobulins (IgA, IgG, and IgM), albumin, and globulin levels).

Statistical analysis

Data were analyzed using SPSS version 19.0 (IBM Corp., Armonk, NY). Continuous variables (e.g., age, CRP, and WBC) were expressed as mean ± standard deviation (SD) or median (interquartile range), while categorical variables were presented as percentages. Group comparisons were performed using an independent t-test for normally distributed continuous variables and χ² (Chi-square) test for categorical variables. Multivariate logistic regression analysis was used to identify independent risk factors for pertussis (delayed/non-immunization status as primary exposure variables). Statistical significance was set at p < 0.05.

## Results

**Figure 1:**
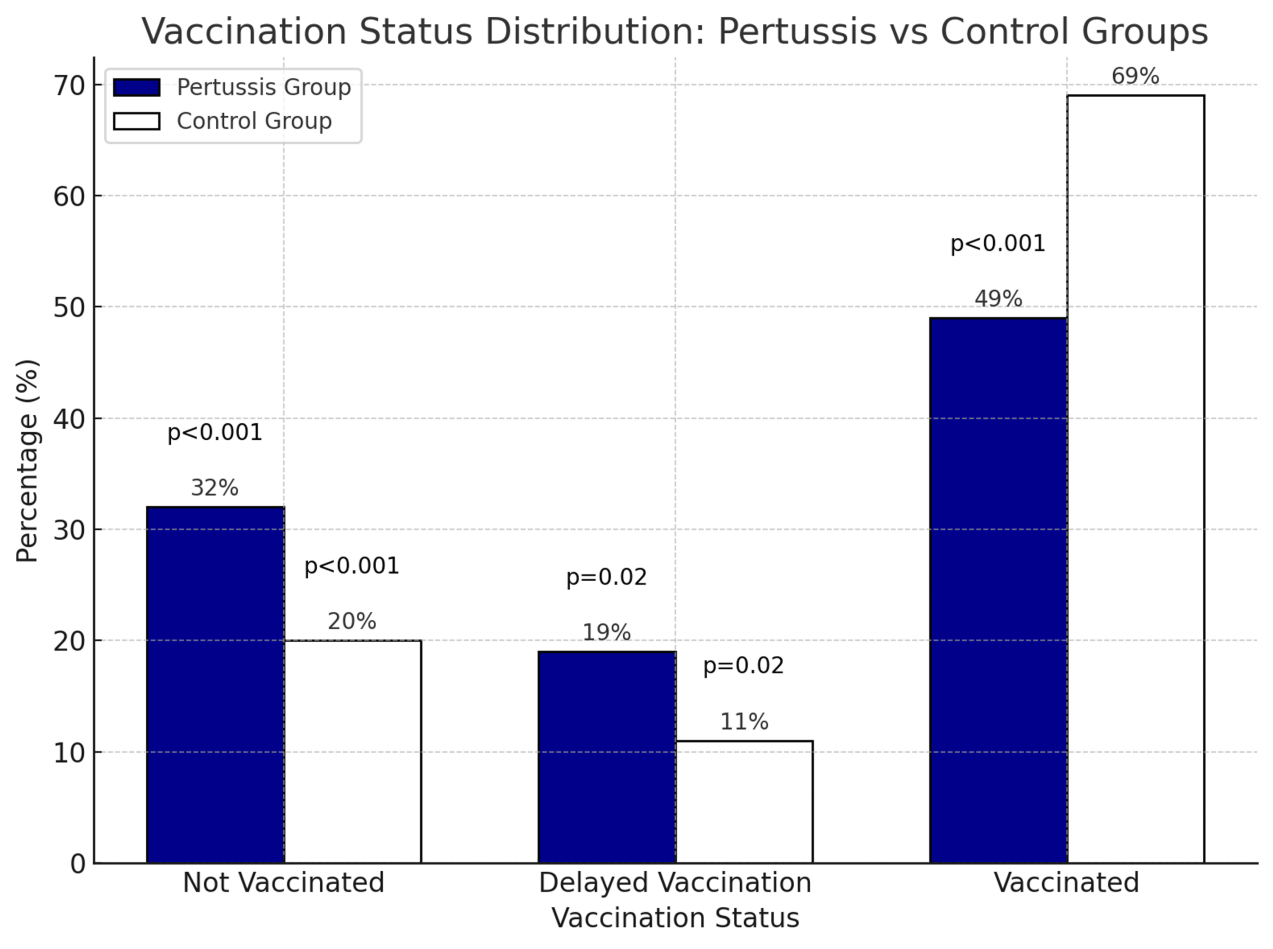
Vaccination status distribution: pertussis versus control groups

The clinical and laboratory characteristics of the pertussis and control groups are summarized in Table 1. The mean length of hospital stay (11.5 ± 4.9 days versus 6.9 ± 5.2 days, p < 0.001), C-reactive protein (CRP) levels (29 ± 7.5 mg/L versus 9 ± 3.6 mg/L, p < 0.001), and white blood cell (WBC) count (31.2 ± 9.7 × 10⁹/L versus 18.2 ± 6.3 × 10⁹/L, p < 0.001) were significantly higher in the pertussis group compared to the control group. Conversely, serum IgM (0.72 ± 0.5 g/L versus 0.98 ± 0.8 g/L, p = 0.04), albumin (38.7 ± 4.7 g/L versus 48.4 ± 5.2 g/L, p < 0.01), and globulin levels (14.8 ± 5.7 g/L versus 19.7 ± 5.1 g/L, p = 0.03) were significantly lower in the pertussis group. Unvaccinated children (32/100, 32% versus 20/100, 20%, p < 0.001) and those with delayed vaccination (19/100, 19% versus 11/100, 11%, p = 0.02) were disproportionately represented in the pertussis group (Figure 1). Table 2 shows the monthly distribution of pertussis cases. Parental concerns about vaccine-related side effects (e.g., fever and local reactions) were the primary reason for delayed or missed vaccinations.

**Table 1:** Demographic, vaccination, and laboratory parameters of all study participants Data presented as mean ± SD or number (%) Statistical tests: independent t-test for continuous variables and χ² test for categorical variables Significance value: p < 0.05 *Significant at p < 0.05 NS: not significant, SD: standard deviation, CI: confidence interval

Parameter	Pertussis group (n = 100)	Control group (n = 100)	t/χ²-value	p-value
Male (number (%))	58 (58%)	57 (57%)	0.016	NS
Age (months) (mean ± SD (95% CI))	7 ± 3.2 (6.4-7.6)	6.9 ± 4.1 (6.1-7.7)	1.202	NS
Not vaccinated (number (%))	32 (32%)	20 (20%)	9.613	<0.001*
Delayed vaccination (number (%))	19 (19%)	11 (11%)	8.314	0.02*
Length of hospital stay (days) (mean ± SD (95% CI))	11.5 ± 4.9 (10.5-12.5)	6.9 ± 5.2 (5.9-7.9)	-10.691	<0.001*
C-reactive protein (mg/L) (mean ± SD (95% CI))	29 ± 7.5 (27.5-30.5)	9 ± 3.6 (8.3-9.7)	32.133	<0.001*
White blood cells (×10⁹/L) (mean ± SD (95% CI))	31.2 ± 9.7 (29.3-33.1)	18.2 ± 6.3 (17.0-19.4)	-6.126	<0.001*
IgM (g/L) (mean ± SD (95% CI))	0.72 ± 0.5 (0.62-0.82)	0.98 ± 0.8 (0.82-1.14)	2.174	0.04*
Albumin (g/L) (mean ± SD (95% CI))	38.7 ± 4.7 (37.8-39.6)	48.4 ± 5.2 (47.4-49.4)	-2.627	<0.01*
Globulin (g/L) (mean ± SD (95% CI))	14.8 ± 5.7 (13.7-15.9)	19.7 ± 5.1 (18.7-20.7)	2.902	0.03*

**Table 2:** Monthly distribution of pertussis cases

Month	Number of cases (%)
January	18 (18%)
February	13 (13%)
March	11 (11%)
April	7 (7%)
May	5 (5%)
June	3 (3%)
July	4 (4%)
August	6 (6%)
September	8 (8%)
October	9 (9%)
November	7 (7%)
December	9 (9%)

Multivariate logistic regression analysis confirmed that delayed vaccination (adjusted odds ratio (OR) = 0.766, 95% confidence interval (CI): 0.545-0.989, p = 0.03) and non-immunization (adjusted OR = 0.744, 95% CI: 0.523-0.964, p = 0.005) were independent risk factors for pertussis (Table 3).

**Table 3:** Multivariate logistic regression analysis of pertussis risk factors Statistical test: logistic regression Significance value: p < 0.05 OR: odds ratio, CI: confidence interval

Factor	β	SE	t-value	p-value	Adjusted OR	95% CI
Not vaccinated	0.517	0.183	7.234	0.005	0.744	0.523-0.964
Delayed vaccination	0.389	0.199	4.638	0.03	0.766	0.545-0.989

## Discussion

This study provides valuable insights into the clinical characteristics and risk factors associated with pertussis in children. Our findings highlight several important clinical features and risk factors that can facilitate the early identification and prevention of this highly contagious disease. The findings of this study underscore the critical role of timely immunization in mitigating pertussis severity and highlight specific biomarkers that may aid in early diagnosis and risk stratification. These insights align with global efforts to combat the resurgence of pertussis, particularly in regions with suboptimal vaccination coverage.

Clinical features and inflammatory markers

Our results demonstrate that children with pertussis exhibit significantly higher CRP levels and WBC counts and prolonged hospitalization compared to controls. These observations corroborate prior studies documenting systemic inflammation as a hallmark of *Bordetella pertussis* infection. The elevated CRP levels reflect the acute-phase response triggered by bacterial toxins, particularly pertussis toxin (PT), which activates pro-inflammatory pathways via Toll-like receptor signaling and cytokine release (e.g., interleukin-6 (IL-6) and tumor necrosis factor-alpha (TNF-α)) [1-3]. Similarly, leukocytosis driven by PT-mediated lymphocytosis is a well-documented phenomenon in pertussis, with WBC counts exceeding 30 × 10⁹/L, often correlating with disease severity and complications such as pulmonary hypertension [4]. The increased length of hospital stays in the pertussis group likely reflects the need for more intensive management in cases of severe illness, which has been previously documented in previous studies, particularly among younger children who may require intensive care for respiratory failure or apneic episodes [1,5,6]. Notably, while CRP and WBC are nonspecific markers of infection, their elevation in pertussis cases supports their utility in differentiating pertussis from other respiratory illnesses, especially in resource-limited settings where molecular diagnostics are unavailable. However, these markers should be interpreted with caution, as overlapping values may occur in cases of co-infection (e.g., viral pneumonia). Future studies could explore the predictive value of combining CRP/WBC with clinical criteria (e.g., paroxysmal cough and post-tussive emesis) to enhance diagnostic accuracy [1,4].

Immunological dysregulation in pertussis

Our study identified significant differences in immune-related laboratory parameters between children with pertussis and those without. Specifically, children diagnosed with pertussis exhibited significantly lower serum IgM, albumin, and globulin levels, indicating a disruption in both immune and nutritional status.

The observed hypoalbuminemia may result from systemic inflammation (as evidenced by elevated CRP), increased vascular permeability due to pertussis toxin, or a catabolic state caused by prolonged illness and poor oral intake. These factors may collectively contribute to reduced albumin synthesis and availability, which could serve as a marker of disease severity and poor prognosis.

Regarding immune function, this study found that IgM, albumin, and globulin levels were significantly lower in the pertussis group. The low serum globulin level in infants and young children is often physiological and has little clinical significance; however, low IgM levels in infancy contribute to susceptibility to infection with Gram-negative bacteria [7]. Therefore, the diagnostic significance of low IgM levels in children with pertussis needs to be explored. The findings of this study suggest that pertussis infection compromised the immune system, impairing the production of key immune proteins. This is particularly important as it highlights the vulnerability of children with pertussis, potentially rendering them more prone to other infections. Previous studies have shown similar patterns of immunological dysregulation in patients with pertussis [1]. This finding aligns with experimental models demonstrating that *B. pertussis* impairs B-cell differentiation and immunoglobulin synthesis via PT-induced disruption of lymphoid follicle architecture [8]. The observed hypoalbuminemia further reflects systemic inflammation and catabolic stress, as albumin synthesis is downregulated during acute-phase responses [9]. Clinically, these deficits may contribute to prolonged recovery and increased morbidity, necessitating close monitoring of nutritional status and immune function in affected children [8].

Interestingly, IgA and IgG levels did not differ significantly between groups, contrasting with studies that report IgA deficiency as a risk factor for severe pertussis [7]. This discrepancy may stem from age-related immune maturation differences, as our cohort included children under six years old, where IgA production remains immature. Longitudinal assessments of immunoglobulin dynamics post-infection could clarify their role in immunity and recovery.

Vaccination status as a modifiable risk factor

According to Egypt's current DTP immunization strategy, vaccinations are administered at two, four, and six months, followed by a booster at 18 months of age. This study identified delayed immunization and non-immunization with the DTP vaccine as significant risk factors for pertussis. Children who were unvaccinated or experienced delays in their immunization schedule were nearly twice as likely to develop pertussis compared to fully vaccinated peers. This corroborates the well-established role of vaccination in preventing pertussis and reinforces the need for timely and complete vaccination in young children to reduce their risk of infection [1]. Research has demonstrated that the efficacy of a single dose of the DTP vaccine (46%) is considerably inferior to the protective effect of three doses (91.7%). The lower effectiveness of a single dose (46%) underscores the importance of completing the full immunization series by six months of age, as recommended by the WHO [10]. Parental concerns about vaccine safety emerged as the primary driver of delayed immunization. Misinformation, logistical barriers (e.g., clinic accessibility), and historical distrust in healthcare systems further compound vaccine hesitancy. Strengthening community engagement through culturally tailored education campaigns, emphasizing vaccine safety and herd immunity, could mitigate these disparities [11].

Public health implications and global context

Despite widespread vaccination, the resurgence of pertussis in recent decades highlights critical challenges in global immunization strategies. Factors such as waning immunity from acellular vaccines, the antigenic divergence of circulating *Bordetella pertussis* strains, and underreporting of mild cases in adolescents and adults perpetuate transmission, creating reservoirs of infection that disproportionately affect unvaccinated or under-immunized infants [1,3]. Our findings underscore the urgent need to address vaccine hesitancy and inequities in routine immunization access, particularly in high-risk populations such as Upper Egypt and other developing countries [12].

Infants who remain unvaccinated or experience delays in completing the DTP series are at heightened risk of severe pertussis due to the absence of vaccine-induced immunity. This vulnerability is exacerbated by the immunological immaturity of young children, as evidenced by lower IgM levels in our cohort, which may further impair pathogen clearance. The World Health Organization (WHO) mandates that all countries achieve ≥90% coverage of three DTP doses by six months of age to protect neonates and young infants, who bear the highest burden of pertussis-related morbidity and mortality [11].

Strengthening immunization programs requires multifaceted interventions, including culturally tailored education campaigns to dispel vaccine myths, expanding mobile vaccination units to underserved areas, and integrating digital health tools to track and remind caregivers about immunization schedules. Nursing plays a critical role in the identification, monitoring, and management of pediatric pertussis cases, particularly through clinical data collection, patient care, and supporting vaccination efforts to prevent disease transmission.

Globally, the reemergence of pertussis underscores the need for adaptive strategies, such as enhanced surveillance, revaccination of adolescents and adults to reduce transmission, and research into next-generation vaccines with improved durability and strain coverage. Addressing these challenges will help healthcare systems reduce the global burden of pertussis and protect vulnerable pediatric populations.

Study limitations

Our study has several limitations that should be considered when interpreting the results. First, as a retrospective medical record review, it is subject to the inherent biases associated with incomplete documentation and missing data. Second, all participants were recruited from a single tertiary care hospital, which may introduce selection bias, as hospitalized patients may represent more severe or atypical cases and may not reflect the broader pediatric population, including those with milder symptoms who do not seek hospital care. Third, although immunization status was verified through health records rather than parental recall, the study did not include a detailed assessment of socioeconomic factors, such as parental education, income level, or geographic access to vaccination centers. The study does not include longitudinal follow-up to assess long-term outcomes or immune recovery post-infection. Lastly, while we observed significant differences in IgM, albumin, and globulin levels between groups, we cannot determine causality or the temporal relationship between these biomarkers and pertussis infection due to the cross-sectional nature of the data.

## Conclusions

In conclusion, this study identifies delayed or incomplete DTP vaccination as a major preventable risk factor for pertussis in children while highlighting specific inflammatory and immunological abnormalities that may inform clinical management. The findings advocate for intensified efforts to improve vaccine coverage through community education, logistical support, and policy reforms. By addressing these challenges, healthcare systems can reduce the global burden of pertussis and protect vulnerable pediatric populations from severe complications.
